# Informed Consent for Intraoperative Neural Monitoring in Thyroid and Parathyroid Surgery – Consensus Statement of the International Neural Monitoring Study Group

**DOI:** 10.3389/fendo.2021.795281

**Published:** 2021-12-07

**Authors:** Che-Wei Wu, Tzu-Yen Huang, Gregory W. Randolph, Marcin Barczyński, Rick Schneider, Feng-Yu Chiang, Amanda Silver Karcioglu, Beata Wojtczak, Francesco Frattini, Patrizia Gualniera, Hui Sun, Frank Weber, Peter Angelos, Henning Dralle, Gianlorenzo Dionigi

**Affiliations:** ^1^ Department of Otorhinolaryngology-Head and Neck Surgery, Kaohsiung Medical University Hospital, Kaohsiung Medical University, Kaohsiung, Taiwan; ^2^ Center for Liquid Biopsy and Cohort Research, and Faculty of Medicine, College of Medicine, Kaohsiung Medical University, Kaohsiung, Taiwan; ^3^ Department of Otolaryngology, Harvard Medical School, Boston, MA, United States; ^4^ Department of Endocrine Surgery, Third Chair of General Surgery, Jagiellonian University Medical College, Krakow, Poland; ^5^ Department of Visceral, Vascular and Endocrine Surgery, University Hospital Halle, Martin-Luther-University, Halle-Wittenberg, Germany; ^6^ Department of Otolaryngology, E-Da Hospital, School of Medicine, College of Medicine, I-Shou University, Kaohsiung, Taiwan; ^7^ Department of General, Minimally Invasive and Endocrine Surgery, Wroclaw Medical University, Wroclaw, Poland; ^8^ Department of Surgery, Ospedale di Circolo, ASST, Settelaghi, Varese, Italy; ^9^ Forensics Division, Department of Biomedical and Dental Sciences and Morphofunctional Imaging, University of Messina, Messina, Italy; ^10^ Division of Thyroid Surgery, China-Japan Union Hospital of Jilin University, Changchun, China; ^11^ Department of General, Visceral and Transplantation Surgery, University of Duisburg-Essen, Essen, Germany; ^12^ Department of Surgery and MacLean Center for Clinical Medical Ethics, The University of Chicago, Chicago, IL, United States; ^13^ Division of Surgery, Istituto Auxologico Italiano IRCCS, Milan, Italy; ^14^ Department of Pathophysiology and Transplantation, University of Milan, Milan, Italy

**Keywords:** intraoperative neural monitoring, thyroid surgery, parathyroid surgery, informed consent, shared-decision making, international neural monitoring study group, voice

## Abstract

In the past decade, the use of intraoperative neural monitoring (IONM) in thyroid and parathyroid surgery has been widely accepted by surgeons as a useful technology for improving laryngeal nerve identification and voice outcomes, facilitating neurophysiological research, educating and training surgeons, and reducing surgical complications and malpractice litigation. Informing patients about IONM is not only good practice and helpful in promoting the efficient use of IONM resources but is indispensable for effective shared decision making between the patient and surgeon. The International Neural Monitoring Study Group (INMSG) feels complete discussion of IONM in the preoperative planning and patient consent process is important in all patients undergoing thyroid and parathyroid surgery. The purpose of this publication is to evaluate the impact of IONM on the informed consent process before thyroid and parathyroid surgery and to review the current INMSG consensus on evidence-based consent. The objective of this consensus statement, which outlines general and specific considerations as well as recommended criteria for informed consent for the use of IONM, is to assist surgeons and patients in the processes of informed consent and shared decision making before thyroid and parathyroid surgery.

## Introduction

Over the last 20 years, there has been a clear trend in thyroid and parathyroid surgery in the use of intraoperative neural monitoring (IONM) to support identification and dissection of the recurrent laryngeal nerve (RLN), facilitate assessment of vocal cord (VC) function and voice outcomes, neurophysiological research, surgical education and training, and medico-legal issues related to loss of VC function (1–15). According to Dralle et al., 70,000 patients received monitored thyroid and parathyroid surgery per year in Germany (16). The contribution of IONM technology has led over time to significant improvement in quality in neck endocrine surgery (17, 18). Current IONM technology enables the monitoring of the external branch of the superior laryngeal nerve (EBSLN) (19–24), real-time monitoring of RLN function by continuous intraoperative nerve monitoring (C-IONM) (25–32) *via* the vagal nerve, and RLN or EBSLN monitoring during endoscopic/robotic surgeries (33–40).

Research in IONM often oversimplifies neuromonitoring equipment and related technological innovations as mere instruments for optimizing RLN identification and preservation during surgery. IONM should not be just considered as a tool for reduction in RLN injury rates, but rather as technology that affords a more comprehensive understanding of RLN function during surgery. Therefore, more integrated and improved information should be offered to patients before surgery (16, 41). Neuromonitoring studies strongly suggest that informing patients about IONM is not only a good practice, it is indispensable for effective shared decision making between the patient and the surgeon and for promoting productive and efficient use of IONM and non-IONM resources (42).

The International Neural Monitoring Study Group (INMSG) (www.inmsg.org) has been at the forefront of IONM technology and procedural adoption since the introduction of neural monitoring in thyroid and parathyroid surgery. The INMSG acknowledges the important supportive roles of IONM in preoperative planning and patient consent in thyroid and parathyroid surgery. The purposes of this paper are to analyze the impact of IONM on the informed consent process and to discuss the existing evidence-based INMSG consensus on informed consent involving the use of IONM.

The studies included in this review were retrieved by a comprehensive Medline search for articles pertaining to informed consent in thyroid surgery. The search was expanded by adding related texts and articles developed from reference lists, personal contacts, conference proceedings, and co-author bibliographies. Focused Medline searches regarding specific aspects of IONM informed consent were done as needed to address any gaps in knowledge. This consensus statement also outlines general and specific considerations and distinguishes essential recommended standard elements of informed consent to IONM to assist surgeons in the clinical decision-making process for the surgical management of thyroid and parathyroid disease. 

## General Considerations

### International Comparison of IONM Availability and Cost

**Figure 1:**
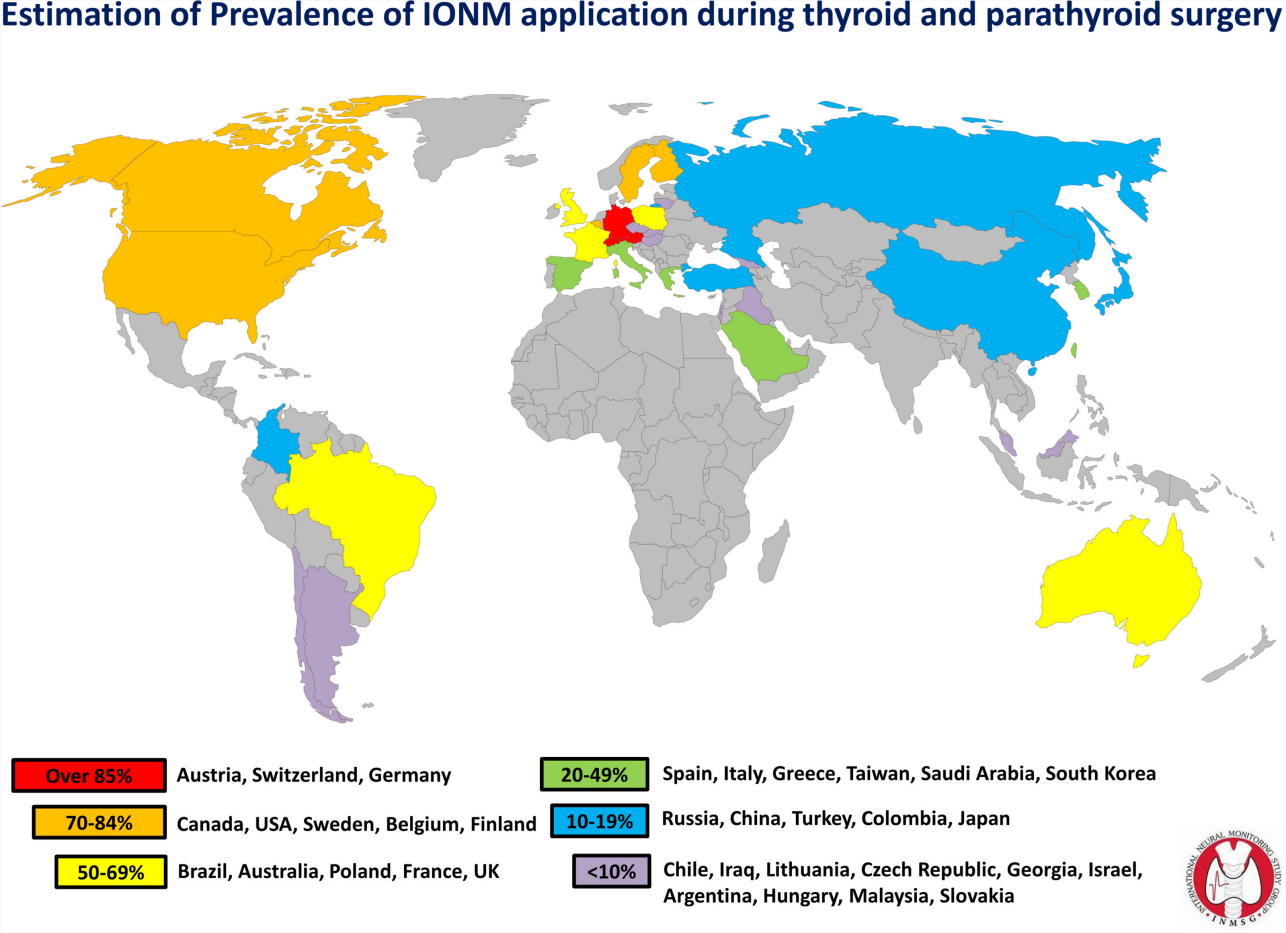
IONM use estimation by country (Source: First World Congress Of Neural Monitoring In Thyroid And Parathyroid Surgery. 17-19, September 2015, Kraków, Poland. http://ionmworldcongress.com/- courtesy of Inomed Medizintechnik GmbH, Emmendingen Germany. World map by www.freeworldmaps.net.).

International comparisons are needed to track IONM use in different healthcare systems, to highlight areas of strength and weakness, and to identify factors that may impede or accelerate adoption of this technology (43–45). The introduction and adoption of IONM varies among different health organizations and different regions (**Figure 1**). For example, while IONM is common in Germany, where it is utilized by over 70% of surgeons, it is utilized in only in a minority of cases in the UK (24%) and Italy (14%) (5, 46–48). In the United States of America (USA), where data for IONM use are widely available, IONM use is increasing (6, 9). Surveys of the American Association of Endocrine Surgeons members found an increase in use of IONM from 7% to 37% between 2001 and 2007 (9). Surveys have also revealed disparities in the distribution of IONM use throughout the USA; e.g., most (69%) of surgeons who use IONM practice in the Northeastern USA (6). In other countries, particularly developing countries, access to IONM technology remains limited (43).

Several factors influence the adoption of IONM technology. High cost of IONM equipment is perhaps the greatest barrier to IONM adoption, particularly in publicly funded healthcare institutions (43). IONM costs vary widely by country and reach as high as €800 per procedure (44), and the consumable medical supplies for IONM may be self-pay items in some regions. Thus, depending on the country and its healthcare system, the technology may be deemed unaffordable.

The wide variation in IONM availability and cost apparently results from intrinsic differences in healthcare systems (i.e., public *versus* private) and differences in health insurance coverage (43). These differences inevitably contribute to non-uniform modalities and structures of informed consent and patient information. For this reason, IONM cost and availability issues mentioned above should be carefully considered when obtaining informed consent. Additional international research is needed to understand better the relationships among IONM strategies, health-care organizations, and the informed consent process.

### Sources of IONM Information

Patients obtain health information from various sources (e.g., TV, radio, newspaper, magazines, the Internet, and personal contacts) to supplement the information provided by their healthcare professionals (49, 50). The time and manner in which individuals use supplemental information depends on various socioeconomic factors, including race, education, income, health literacy, and health status (49, 50).

Patients as well as relatives/friends of patients use the Internet as a source of information about an illness, including therapies, side effects, and new surgical procedures. According to data from an Italian survey, 6 out of 10 internet users considered internet searches an acceptable substitute for consultation with family doctors (50). In the 2,300 online questionnaires completed in this survey, 58% of the respondents replied that when they experienced a health problem, they first searched for information online. Demographic characteristics associated with use of the Internet to search for health-related news and information include female gender, young age, and medium to high socio-economic level.

Since traditional media (i.e., television, radio, etc.) provide limited information about IONM, in 2015 INMSG established a website specifically for providing IONM information (www.inmsg.org). Nevertheless, Ferrari C. et al. reported that, for the general public, internet information about IONM during thyroid surgery, is too specific, too difficult to understand, and too difficult to access (51). The authors analyzed IONM-related websites available to the general public that specifically discussed thyroid surgery. Most websites (64%) were associated with scientific publications. Most websites (91%) were in English, and only 19% of the websites provided multilingual information (including English) or in were written in other languages. The authors rated 58% of the sites as “excellent-to-good” and 42% as “fair-to-poor”. The median Flesch Reading Ease Score was 49.6; the median Flesch-Kincaid Grade Level was 13.85 (51). Internet information regarding IONM thus is not only inadequate for properly educating patients, but potentially misleading as most websites tend to present only the benefits of the technology. Patients who would like to understand IONM technology need improved access and quality of online resources and information on IONM. Treating physicians also serve as another valuable source of IONM information for patients and are expected to provide detailed and reliable information and counseling in the benefits as well as limitations of current IONM systems (50).

### IONM Curriculum and Responsibility for Monitoring

**Figure 2:**
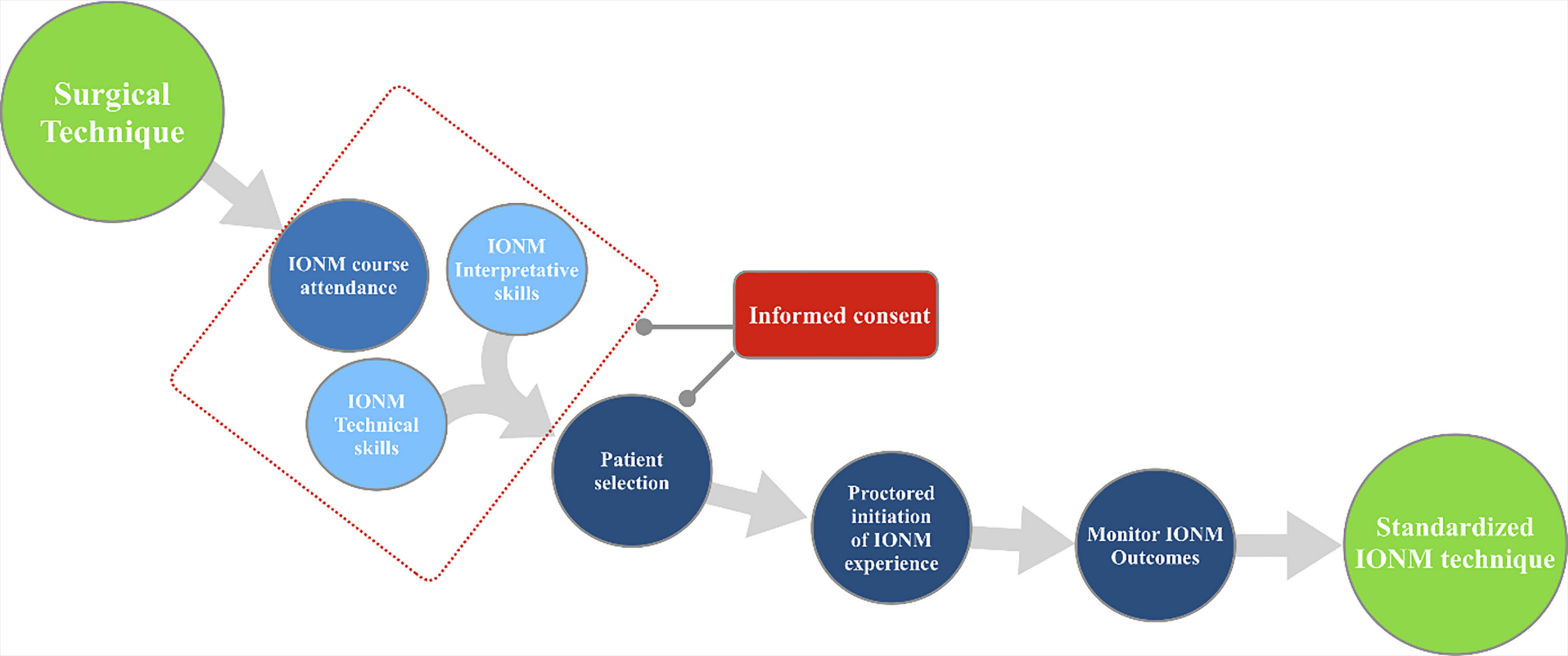
Standardization of IONM during thyroid and parathyroid surgery. IONM specific informed consent is an essential topic to be discussed during formal INMSG courses. Informed consent must be offered to all patients in whom IONM is utilized.

Before implementing IONM in practice, surgeons should have completed relevant surgical training and should be able to address common complications (1, 4, 52). IONM procedures are maximized with a well-trained team. As routine use of IONM increases, surgeons should include nerve monitoring courses in their curriculum to ensure competency (**Figure 2**).

The need for ongoing professional education training to acquire and maintain knowledge and expertise in current IONM technologies and applications will also contribute to progressive improvement of pre-operative information given to patients. Patients must be adequately informed of the experience of the surgeon and/or surgical team in performing the proposed surgical intervention. Surgeons have four main responsibilities in an IONM-assisted surgical procedure (**Figure 2**):

#### (A) Technical Responsibilities

Using and setting up IONM equipment correctly and understanding the inherent properties of the system to avoid inaccurate baseline setup (e.g., confirming proper muscle relaxation type/dosage, correct electrode placement with low impedance, etc.) (1).

#### (B) Interpretive Responsibilities

Surgeons should be able to distinguish between true and artifactual responses and perform appropriate troubleshooting to identify and correct the issue at hand (52). 

#### (C) Information Responsibilities

The surgeon has a duty to provide patients with accurate and relevant IONM information, including its benefits and limitations. When providing information to the patient, the surgeon must consider many factors, including education level, emotional state, and ability to understand the content of the discussion. In all cases, further information requests by the patient must be satisfied. However, information related specifically to IONM technology may be limited to those elements that the patient’s culture and psychological condition are able to understand and accept, to avoid superfluous clarification and discussion of data and technical aspects of the procedure which are not of benefit to the patient.

#### (D) Documentation Responsibilities

The surgeon is responsible for carefully reviewing and documenting the information, including patient’s thyroid history, preoperative radiologic imaging, pre-operative symptoms, pre- and postoperative laryngeal examination, detailed surgical or operative report with intraoperative RLN findings, and surgical complications if any.

Electromyographical (EMG) muscle action potential recording and documentation is possible with all modern IONM devices (1, 10). IONM converts muscle activity into recorded EMG signals, which are often possible to print out (1). Such information provides the surgeon with a summary of relevant information collected during the operation (i.e., artifact signal, amplitude decline, and latency increase) and may be of interest for patients seeing to understand the use of IONM (1, 10, 16). Documentation of IONM use during thyroid and parathyroid surgery can include time-traceable measures of EMG amplitude, latency, waveform morphology, and magnitude of stimulating current. EMG curves provide proof of intact nerve function.

Monitoring nerves during surgical procedures may potentially reduce the medico-legal liability of the surgeon as well as the economic losses of the patient, healthcare system, and insurance companies. Recorded nerve signals are also important for early determination of whether voice changes are RLN-related (1, 4, 10, 16). From a medico-legal perspective, recording and documenting the muscle action potential is recommended at the beginning (V1; R1) and at the end of the resection (V2; R2) for each side of resection. Additionally, documentation included in the medical record should, at minimum, include V2 stimulation after thyroidectomy is completed on the first side, which indicates that thyroidectomy can safely proceed on the contralateral side (13, 46, 53, 54).

### Is IONM Evidence-Based?

Evidence of the benefit of IONM is limited to class II and class III studies, the same evidence level generally applied to other technologies and clinical practices in thyroid surgery (52, 55, 56). In studies that have investigated IONM use in other surgical procedures (including spinal surgery, vascular surgery, and brain surgery), even highly accredited investigations (e.g., Cochrane reviews) have not provided class I evidence that IONM improves safety (57, 58). Meta-analyses have reported no conclusive evidence of the superiority or inferiority of IONM over visual nerve identification for any outcome measures (52, 59–65) (**Table 1**).

**Table 1:** Summary of meta-analysis articles on the topic of RLN palsy after thyroid surgery with and without use of IONM.

Author	Year	Journal	Studies included in meta-analysis	Bibliographic database	NAR	T. Palsy with IONM (%)	P. Palsy with IONM (%)	T. Palsy without IONM (%)	P. Palsy without IONM (%)	*p* value	Conclusion
Dralle H	2008	World J Surg (52)	6 with and without IONM	Medline	19290 with IONM 6671 without IONM	2.7	0.8	2.8	0.9	0.45 (T.) 0.30 (P.)	IONM does not result in lower postoperative RLN palsy rates compared with RLN dissection alone. Visual identification remains the basis for nerve protection.
			10 with IONM only	Medline	7374 with IONM	3.7	0.7	NA	NA	NA	
Rulli F	2014	Acta Otorhinolaryngol Ital (64)	8	PubMed and Ovid, and the Cochrane Library database	Total 5257	NA	NA	NA	NA	0.035 (T.) 0.235 (P.)	IONM prevents transient injury. No advantage was found in permanent injuries.
Pisanu A	2014	J Surg Res (65)	20	Embase, Medline, Cochrane, PubMed, and Google Scholar databases	24038 with IONM 11475 without IONM	2.62	0.79	2.72	0.92	0.552 (T.) 1.000 (P.)	Using IONM or not showed no statistically significant difference in the incidence of RLN palsy.
Lombardi CP	2016	Surgery (63)	14 (4 RCTs)	PubMed, Scopus, and CENTRAL	25814 with IONM 15929 without IONM	NA	0.7	NA	0.9	0.071 (P.)	IONM does not prevent permanent nerve palsy.
Sun W	2017	Clin Endocrinol (60)	9	PubMed, SCIE and Wan Fang databases	1109 with IONM 1327 without IONM	3.98	1.26	6.63	2.78	0.227 (T.) 0.031 (P.)	Significant effect of IONM in preventing permanent RLN palsy.
Yang S	2017	Inter J Surg (61)	24 (4 RCTs)	PubMed, Embase, and the Cochrane library	8668 with IONM 8535 without IONM	1.82	0.67	2.58	1.07	<0.001 (T.) 0.005 (P.)	Benefits of reducing RLN palsy rate by using IONM. Using IONM may improve the outcome by reducing amount of residual thyroid tissue.
Wong KP	2017	Inter J Surg (62)	10	Pubmed, Medline, Embase and CENTRAL	6155 with IONM 4460 without IONM	2.4	1.3	3.9	1.6	0.016 (T.) 0.104 (P.)	Use of IONM during high-risk thyroidectomy decreases the rate of RLN palsy. IONM should be recommended during re-operation or thyroidectomy for malignancy.
Cirocchi R	2019	Cochrane Database Syst Rev (59)	5 RCTs	CENTRAL, Medline, Embase, ICTRP Search Portal and ClinicalTrials.gov	1451 with IONM 1444 without IONM	2.2	0.7	3.6	0.9	0.09 (T.) 0.54 (P.)	No evidence for the superiority or inferiority of IONM over visual nerve identification alone on any of the outcomes measured.

RLN, recurrent laryngeal nerve; IONM, intraoperative nerve monitoring; NAR, nerves at risk; T. palsy, Transient RLN palsy; P. palsy, Permanent RLN palsy; RCT, randomized controlled trial; CENTRAL, Cochrane Central Register of Controlled Trials; NA, not assessable.

Class I studies in the use of IONM in thyroid surgery are not possible for at least two reasons (52, 55–65). First, the likelihood of IONM use in preventing a transient RLN deficit is so low that a controlled study that randomly assigned patients to a control group or a monitored group would be arduous (52). Moreover, the incidence of permanent RLN complications is even lower. Thus, IONM use would be aimed at further reducing the incidence of a complication that already has a low incidence (52). An adequately powered study would be laborious as the number of patients needed would likely exceed the number of patients possible to enroll in multi-institutional studies (52).

Accordingly, future perceptions of the benefit of IONM will continue to be based on its good clinical outcomes, historical control studies, and cost-benefit evaluations (66). Therefore, in our opinion during the informed consent process, telling patients that IONM is adequately evidence-based in reducing RLN paralysis is misleading and unethical. 

### IONM and Malpractice Claims of Nerve Palsy

Malpractice claims related to thyroid and parathyroid surgery are costly and time-consuming (67). All permanent and transient consequences of thyroid surgery constituted malpractice claims and the RLN injury or palsy is the leading cause (10, 67–69). Dralle et al. (10) reported nearly 60% of 75 malpractice claims between 1995 and 2010 involved RLN palsy (21 unilateral and 22 bilateral), with a 45% tracheostomy rate for bilateral palsy. They noted that IONM has become the subject of pleading in 4 of 7 malpractice claims involving unilateral or bilateral RLN palsy since 2007. In none of these cases did IONM follow international standards, resulting in 3 plaintiff verdicts. In addition, Gartland et al. (67) found that bilateral RLN injury, accounting for up 18% of 128 malpractice suits in the US, was predictive of plaintiff verdicts (OR 3.58, P=0.03) on multivariable regression analysis.

The growing appreciation that standardized IONM can prevent bilateral RLN palsies after signal loss on the initial side of resection may become increasingly relevant to malpractice litigation (10). An informed consent detailing the strengths and weaknesses of IONM, including the need to change operative treatment plans in the event of LOS, may serve as a line of defense in the event of litigation (11, 70, 71).

## Defining the Standard of IONM Informed Consent

### Purpose of Informed Consent in Thyroid Surgery

Before undergoing IONM-assisted thyroid surgery, the patient must be adequately informed of the purpose and nature of the endocrine intervention as well as its potential benefits and risks (72). A dedicated form for written informed consent for thyroid or parathyroid surgery should include the following information: type of surgery, objectives of surgery, consequences of thyroidectomy or parathyroidectomy, risk and benefits of declining thyroidectomy or parathyroidectomy, alternative procedures (active surveillance, thermal ablation, etc.), and possible risks of thyroidectomy or parathyroidectomy (73, 74). The extent of informed consent for IONM-assisted thyroidectomy or parathyroidectomy depends on the purpose and context (i.e., legal, ethical, administrative, documentation and knowledge).

### Target Population and Timing

Informed consent must be obtained from a patient (or appropriate guardian or healthcare proxy) referred for IONM-assisted thyroid surgery. Whenever possible, informed consent should be obtained from the patient (or guardian) well in advance of the intervention to allow for adequate time for reflection. Ideally, the patient should be briefed on IONM in the planning stage of the intervention (75).

### Counseling Specifically Related to IONM

**Figure 3:**
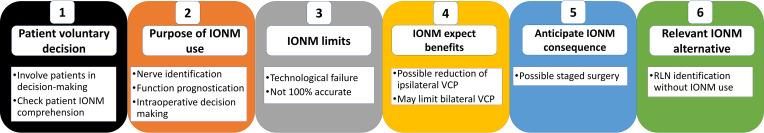
IONM Informed Consent. Diagram showing key information of the preoperative encounter and IONM informed consent process. It is necessary to document the parties involved in the informed-consent process.

Although the criteria for adequate informed consent may differ by country and by hospital, in our opinion, the consent process should always include discussion of (a) IONM limitations (IONM accuracy, technical failure, etc.) and (b) IONM consequences (i.e. possible staged thyroidectomy) (76–78) (**Figure 3**).

#### IONM Limitations

As with any intraoperative technology, we believe it is important to explain to the patient as part of informed consent that the IONM has limitation and may fail. IONM false-positive rates, surgeon IONM inexperience, and technical failures are the main reasons IONM may provide unreliable results (1, 2). According to the literature, device malfunction or false IONM results may occur in 1% to 13% of procedures (1, 2, 79, 80).

#### Staged Thyroidectomy

Before consenting to bilateral thyroidectomy with IONM, the patient should be advised that a staged thyroidectomy may be needed. As IONM use increases, an adaptation of the staged strategy will be necessary. The patient should be advised that a such a staged strategy is neuromonitoring-dependent. In the event of intraoperative signal failure during the first operated side, a 2-step procedure is advised in order to avoid the catastrophic effects of bilateral RLN paresis (74).

Pre-surgery planning for the second side in cases of prior aborted total thyroidectomy or bilateral parathyroidectomy should include three surgical options for addressing the contralateral side with intact RLN function:

(1) No contralateral resection at initial surgery with LOS in cases of bilateral goiter, Graves’ disease, or low risk thyroid carcinoma (differentiated and medullary thyroid carcinomas) with the aim of 2-stage completion surgery after verification of recovery of nerve function on the initial side.
(2) Contralateral subtotal lobe resection keeping the dissection plane ventral to the RLN plane in cases of benign goiter thus maintaining a safety distance to the nerve with the aim of avoiding a second operation, because of patient´s co-morbidities.
(3) Total thyroidectomy as planned for advanced thyroid carcinomas (including undifferentiated thyroid carcinomas) with the aim of immediate postoperative radioactive iodine therapy.

### IONM Informed Consent Documentation

As in all complex medical technologies, the possibility of technological failure should be discussed with the patient pre-operatively during informed consent. As noted above, the surgeon should also discuss that a staged thyroidectomy might be indicated if the initial side shows evidence of LOS. The informed consent form should state the limitations of IONM and the procedure for LOS on the first-side during thyroidectomy as follows:

#### For IONM Limitations

“As with all technologies applied in surgery, IONM technology can also fail in accuracy”.

#### For Staged Thyroidectomy

“During thyroid surgery we are using a device to assess the function of the RLN in real-time. When there is a loss of signal and possible loss function of the RLN on the first side of dissection (dominant side), we stop the procedure to possibly prevent bilateral VC palsy. This would result in a possible second surgery or staged thyroidectomy.”

### Practical Implementation Advice: Improving the IONM Informed Consent Process

The informed consent process should only be implemented if the patient has capacity to consent or has an appropriate surrogate decision maker. To ensure that the informed consent discussion is understood by the patient or surrogate, the language used in the discussion should target the appropriate level of health literacy. Surgeons should be prepared to provide additional information when requested by patients and/or surrogates. Some thyroid clinics may opt to provide informational brochures or videos. The use of multimedia technology (e.g., videos of surgical procedures, computer animations, and graphics), in addition to traditional forms of printed or hand-produced material, may reduce inconsistencies in the amount of information assimilated by patients with different education levels and may improve the quality of the informed consent process (81).

## Special Considerations

### EBSLN Monitoring

In addition to RLN paralysis, another important consequence of thyroidectomy is the possible change in voice quality and projection due to EBSLN paresis. The EBSLN is vulnerable to damage in patients with a large goiter, a thyroid tumor of the superior pole, a short neck, or lower lying nerves such as Cernea types 2a and 2b (20, 21, 82). Recent reports indicate that IONM aids in EBSLN identification and preservation in both conventional and endoscopic thyroidectomy (19, 21, 24, 39, 83–85). In the opinion of the authors, the benefits of IONM on EBSLN preservation is still nascent. However, discussion of the potential for EBSLN paralysis may be especially relevant in certain patients, such as voice professionals, where discussion of the utility (and limitations) of EBSLN monitoring may be important, if not required.

### Exception to IONM Informed Consent

The surgeon has a professional responsibility to provide the patient with IONM information that is accurate, relevant and commensurate with the health literacy of the patient. To reiterate, suggesting that IONM use is adequately evidence-based in reducing RLN palsies is inappropriate, since data in the literature are still insufficient to support this claim. We believe that certain issues (particularly technological issues) can be excluded from the IONM informed consent discussion. Examples include:

- The availability, difference and options of IONM systems (86, 87) and recording EMG tube, (i.e. post-cricoid or anterior laryngeal electrodes) (88–94).
- The availability, difference and options of I-IONM stimulating probe or dissectors (34, 40, 95–99).
- The availability, difference and options of C-IONM stimulation electrodes, and the surgical approach of C-IONM placement electrode on the vagus nerve (26, 100–103).

### IONM in the Case of Preoperative Nerve Palsy

Although preoperative vocal cord palsy is often associated with RLN invasion by advanced thyroid malignancy, it may also be associated with benign conditions that result in compression or stretching or in inflammation (Hashimoto’s or Riedel’s infiltration) with a reported incidence of 0.2 to 1% (104–106). A detectable EMG signal in the case of vocal cord palsy may indicate residual neural function in the form of retained electrical conductivity (14). In benign condition, some studies (107, 108) suggest that a short duration of vocal cord palsy increases the probability of postoperative recovery of vocal cord function and that IONM is helpful for mapping a severely displaced and compressed RLN such as in the case of a large substernal goiter (108). In Kamani et al., recognizable RLN electrophysiologic activity was preserved in over 50% of cases with preoperative vocal cord dysfunction. In addition, malignant invasion of the RLN was associated with preoperative vocal cord paralysis in only 50% (109). In Lorenz et al., 41 of 285 patients (14%) with preoperative vocal cord palsy had a detectable EMG signal. If the RLN is preserved during surgery, and if the malignancy has not directly invaded the RLN, functional recovery is reported as high as 38% to 89% (110).

The postoperative outcome of the paralyzed RLN and its management determine what strategy will be appropriate for managing the RLN if contralateral surgery is required (14). Therefore, patients with preoperative vocal cord palsy should be informed of the benefits of IONM. Additionally, patients who consent to use IONM must be adequately informed of surgical strategies for intraoperative RLN management.

### Patients Who Refuse IONM

The surgeon must not perform any diagnostic-therapeutic-surgical procedure without the consent of a validly informed patient. Depending on the country and its healthcare system, the cost of IONM consumable medical supplies may be unaffordable by patients without insurance coverage (111). The surgeon must desist from any IONM use in the case of explicit refusal of IONM by a patient capable of understanding. In their decades of experience in IONM, however, INMSG Board members have seldom encountered a patient who refused IONM.

### In the Event of Unavailable IONM Technology

The IONM technology may be unavailable or not utilized in the following scenarios:

(i). The patient has given written consent to IONM, but the IONM device is currently unavailable or malfunctioned preoperatively at the institution. The thyroid intervention can be rescheduled or referred to another center where IONM is available.
(ii). The patient has signed IONM informed consent, however the device has malfunctioned intraoperatively. In the event of intraoperative IONM device breakdown, the thyroid intervention continues without IONM. This possibility should be discussed preoperatively with the patient (i.e., IONM limitations - see above) and disclosed post-operatively to the patient.

### IONM in Clinical Research

It should be emphasized that large and rapid technological advances in IONM have broadened the framework of possible alternatives in IONM procedures. Therefore, the inherent risks and benefits of the IONM procedure proposed for the patient must be clearly explained and supported with documentation, which may include opinions in the literature on the proposed IONM modality. The experience and case history of the IONM team must also be clearly explained.

For researchers, prior review and approval from the local institutional review board (IRB) or independent ethics committee (IEC) is mandatory before performing clinical research in IONM. The IRB/IEC is responsible for reviewing the research proposal and ensuring that informed consent procedures are adequately and ethically implemented without jeopardizing the rights, safety, and well-being of the human subjects.

## Limitations of the INMSG Consensus

The INMSG, a multidisciplinary international group established in 2006, comprises surgeons, laryngologists, voice and laryngeal electromyography specialists, anesthesiologists, and researchers who have extensive experience in thyroid and parathyroid neural IONM and have previously published multiple manuscripts and guidelines related to RLN and EBSLN monitoring (1, 4, 13–15, 19). Although we hope that this consensus statement identifies high-quality studies that provide a strong quantitative base of evidence for the above recommendations for informed consent to IONM, the resulting bibliography reflects a bias within the literature toward thyroid surgery and much of the quantitative literature on this topic is descriptive in nature. Informed consent is primarily a legal, ethical and administrative concept; although often informed by data, the standards of scholarship in law and ethics focus on the strength of analytical argument rather than the weight of empirical data. Therefore, we sought to synthesize the available knowledge on this subject by referencing empirical data as needed and summarizing relevant arguments that are particularly prevalent, persuasive or insightful.

## Conclusion

Improving voice outcomes after thyroid and parathyroid surgery is an important issue in the quality-of-life era. Surgical use of IONM has gained widespread acceptance in the international community as a useful technique for reducing possible RLN and EBSLN injury in these procedures. This INMSG consensus statement outlines the general and specific considerations regarding the surgical use of IONM and provides essential recommended standard elements of informed consent for the use of IONM thereby assisting surgeons and patients in the informed consent process and in shared decision making prior to thyroid or parathyroid surgery.

## Author Contributions

All authors have made a substantial contribution to the concept of the article, drafted and revised the article critically for important intellectual content. All authors have read and agreed to the published version of the manuscript.

## Funding

This study was supported by grants from Kaohsiung Medical University Hospital, Kaohsiung Medical University (KMUH109-9M44), Kaohsiung Municipal Siaogang Hospital/Kaohsiung Medical University Research Center grants (KMHK-DK(C)110009, I-109-04, H-109-05, I-108-02), and Ministry of Science and Technology (MOST 110-2314-B-037-104-MY2, MOST 110-2314-B-037-120), Taiwan.

## Conflict of Interest

The authors declare that the research was conducted in the absence of any commercial or financial relationships that could be construed as a potential conflict of interest.

## Publisher’s Note

All claims expressed in this article are solely those of the authors and do not necessarily represent those of their affiliated organizations, or those of the publisher, the editors and the reviewers. Any product that may be evaluated in this article, or claim that may be made by its manufacturer, is not guaranteed or endorsed by the publisher.
